# LaSVM-based big data learning system for dynamic prediction of air pollution in Tehran

**DOI:** 10.1007/s10661-018-6659-6

**Published:** 2018-04-20

**Authors:** Z. Ghaemi, A. Alimohammadi, M. Farnaghi

**Affiliations:** 1 Faculty of Geodesy and Geomatics Engineering, K.N. Toosi University of Technology, No. 1346, ValiAsr Street, Mirdamad cross, Tehran, 19967-15433 Iran; 2 GIS Center, Department of Physical Geography and Ecosystem Science, Lund University, Lund, Sweden

**Keywords:** Spatio-temporal, LaSVM, Online prediction, Big data, Urban air quality, Tehran

## Abstract

Due to critical impacts of air pollution, prediction and monitoring of air quality in urban areas are important tasks. However, because of the dynamic nature and high spatio-temporal variability, prediction of the air pollutant concentrations is a complex spatio-temporal problem. Distribution of pollutant concentration is influenced by various factors such as the historical pollution data and weather conditions. Conventional methods such as the support vector machine (SVM) or artificial neural networks (ANN) show some deficiencies when huge amount of streaming data have to be analyzed for urban air pollution prediction. In order to overcome the limitations of the conventional methods and improve the performance of urban air pollution prediction in Tehran, a spatio-temporal system is designed using a LaSVM-based online algorithm. Pollutant concentration and meteorological data along with geographical parameters are continually fed to the developed online forecasting system. Performance of the system is evaluated by comparing the prediction results of the Air Quality Index (AQI) with those of a traditional SVM algorithm. Results show an outstanding increase of speed by the online algorithm while preserving the accuracy of the SVM classifier. Comparison of the hourly predictions for next coming 24 h, with those of the measured pollution data in Tehran pollution monitoring stations shows an overall accuracy of 0.71, root mean square error of 0.54 and coefficient of determination of 0.81. These results are indicators of the practical usefulness of the online algorithm for real-time spatial and temporal prediction of the urban air quality.

## Introduction

Air pollution is considered as one of the most crucial problems in industrial and populated cities. Adverse effects of air pollution on human health have been the subject of many studies (Brunekreef and Holgate 2002; Chan-Yeung 2000; García Nieto et al. 2013) and development of effective techniques for monitoring and prediction of air pollution is of prime importance. Online air pollution forecasting for the next few hours enables decision makers to urge the vulnerable groups to avoid outdoor activities during the risky times. Also, reliable forecasts can provide the required data for an urban air quality analysis and management system. By using this information, decision makers can take proper measures for emission reduction. The existing air quality monitoring stations in urban areas continuously record high volumes of pollutant concentrations. These data need to be effectively utilized for analysis and prediction of the air quality indices.

Air pollution is affected by various factors such as the atmospheric conditions and geographical parameters such as the land use, traffic, elevation, and location (Zheng et al. 2013; Hasenfratz et al. 2012). Therefore, air pollution prediction is regarded as a complex and nonlinear problem (*P. Wang* et al. 2015; Ghaemi et al. 2015). Importance and complexity of urban air pollution prediction problem have led to the development of a wide variety of prediction techniques. These approaches can be classified into the two major categories of deterministic and statistical methods (*P. Wang* et al. 2015). The widely used Gaussian Dispersion Model is one type of deterministic methods. In these models, air quality is predicted by simulating the physical and chemical processes of the atmosphere (Bellander et al. 2001; Ranzato et al. 2012; Venegas et al. 2014; Mansourian et al. 2011). Although dispersion models have been shown to be exact (Finardi et al. 2008), they require reliable information about the sources of the pollutants as well as the physical and chemical characteristics of the atmosphere. Collection of such continuously varying information is quite difficult for large-scale applications. Moreover, employment of these models in real-world problems with huge amount of data is very time-consuming (Chaloulakou et al. 2003; Kumar and Goyal 2011; Zhang et al. 2013). Deficiencies of deterministic models have led the statistical methods to be more popular in real-world problems (Chen et al. 2013). Kriging, regression, and artificial intelligence are examples of statistical models which have been widely applied to model the spatial and temporal variation of the air pollution (Briggs et al. 1997; Jerrett et al. 2001; Su et al. 2007; García Nieto et al. 2013). Among them, artificial intelligence techniques have shown high capabilities for solving the complex and non-linear air pollution problems. Due to their greater flexibility and accuracy, artificial neural networks (ANN) have been widely used in air pollution prediction (Pfeiffer et al. 2009; Kurt and Oktay 2010).Viotti et al. (2002) and Elangasinghe et al. (2014) have utilized neural networks to forecast urban and coastal air quality based on the traffic level along with the weather conditions (Viotti et al. 2002; Elangasinghe et al. 2014). Lack of sufficient number of monitoring stations throughout the cities limits the abilities of air pollution prediction methods to address the spatial distribution of contamination. To overcome this deficiency, Pfeiffer et al. (2009) and Wahid et al. (2013) have proposed a solution based on neural networks that predicts air pollution by combination of the spatial parameters and monitoring station data (Wahid et al. 2013; Pfeiffer et al. 2009). Although neural networks can be used to model nonlinear problems, practical applications of these models particularly in the case of work with big input data, suffer from different drawbacks such as the high computation time, overfitting, local minima and poor generalization abilities (Niska et al. 2004; Singh et al. 2013). Due to their computational efficiencies and generalization abilities (García Nieto et al. 2013; Ip et al. 2010), recently, Support Vector Machines (SVM) have been regarded as the interesting alternative approaches to the conventional statistical models (Lu and Wang 2005; Luna et al. 2014; Yeganeh et al. 2012). As an example, Juhos et al. (2008) used ANN and SVM methods in Szeged to predict the concentration of NO and NO_2_ in high-traffic areas. As compared with ANN, SVM showed more reliable forecasting results (Juhos et al. 2008). Concentrations of CO for the next day have been predicted by combination of the SVM and the Partial Least Square approach (Yeganeh et al. 2012).

Development of data measurement tools such as the air quality monitoring stations and embedded sensors in mobile devices provides various types of data about the urban air quality. Such data are characterized by the extreme volume, wide variety, and high velocity. Therefore, conventional methods cannot typically handle the volume, variety, and velocities associated with the air pollution streaming data. In this respect, the existing typical SVM algorithm is not able to process the huge data that needs frequent and continuous updating. Because, once a typical SVM algorithm is trained, it works as the stationary model afterward and when new training samples are available, learning has to restart again using the whole training samples which have been presented so far (W. Wang et al. 2008). This process is computationally expensive and time-consuming. Online algorithms are regarded as an alternative to the conventional static methods. Because of their capabilities to deal with voluminous and dynamic data, the online algorithms have become popular among the scientists. In this regard, a number of online algorithms based on the SVM have been presented for prediction of the dynamic phenomena such as the air pollution. Wang et al. (2008) applied an online SVM algorithm to predict the time series of pollutant concentrations. Pollutant concentrations and meteorological data were used in this study. Although the tested online SVM showed a good prediction performance, no geographical parameters were used for prediction of the spatial distribution of pollutants (W. Wang et al. 2008).

Main objective of this study is to propose and test a SVM-based online system for prediction of the urban air quality in Tehran, Iran. Pollutant concentrations and meteorological data of Tehran, continuously measured by the monitoring stations, are used as the input data. Also, in order to address the problem of insufficient coverage of monitoring stations throughout the city and model the spatial distribution of pollutant concentrations, some geographical parameters are employed. The proposed algorithm is continually trained based on the streaming data received from the monitoring stations. To overcome the deficiencies of the typical SVM in dealing with the big and streaming data, the online algorithm includes a removal step which eliminates redundant data during the training process. Reduction of the training samples leads to significant reduction in the volume of data required for re-training. The trained algorithm is then able to predict the air quality in each selected location for the next 24 h. Prediction maps are made accessible to the user via an air pollution monitoring and prediction web site. Computation time and accuracy of the online SVM is compared to those of the typical SVM. Experimental results confirm usefulness of the system due to its acceptable accuracy and processing time. The remainder of this paper continues as follows. In material and methods section, the case study, data sets, and data preparation steps are described. The developed online algorithm for real-time air pollution prediction is also presented in this section. Results are discussed in the next section. Finally, the last section concludes the paper and provides the future directions.

## Material and methods

### Case study

**Fig. 1:**
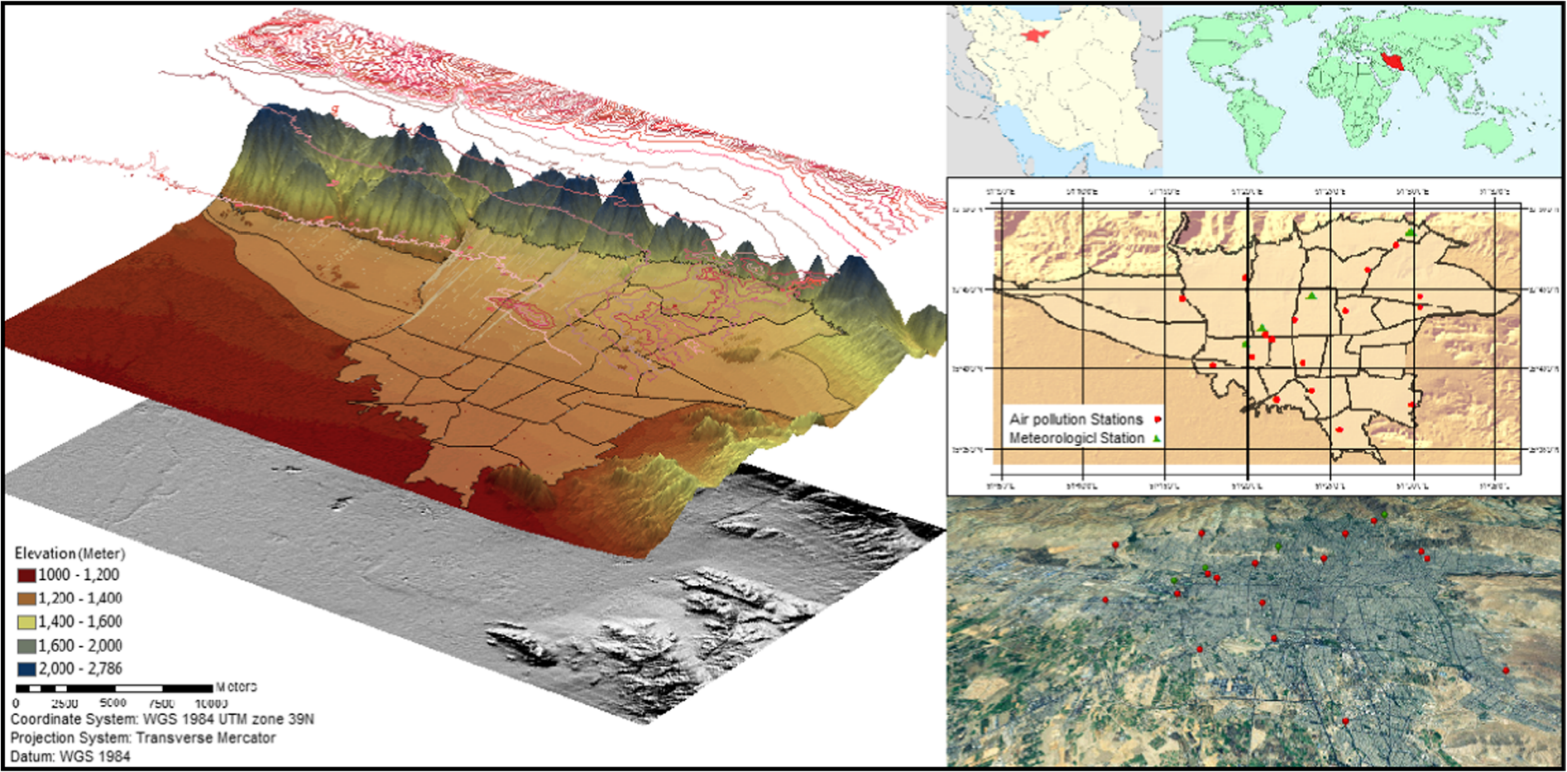
Geographic position of the study area and spatial distribution of the existing air pollution monitoring stations

Tehran, the capital of the Islamic Republic of Iran with approximately 8.5 million inhabitants, is the largest commercial and political center of the country. Tehran is surrounded by the high altitude mountains in the North and a vast desert in the South. Due to the increasing number of vehicles and industrial areas, Tehran suffers from severe air pollution. Figure 1 presents locations of the air pollution monitoring sensors and weather stations throughout the city along with the elevation map of Tehran. The exaggerated elevation map is generated using the elevation data from NASA’s 90 m resolution SRTM data.

### Parameters, dataset, and data preparation

The input parameters used in this study are composed of the pollutant concentrations and meteorological and geographic data. These are briefly described below.

### Pollution data

Hourly air quality data have been collected from the 21 air pollution monitoring stations during the 6 years from 2008 to 2014. These stations record data of some important air pollutants including the *carbon monoxide* (CO), *nitrogen dioxide* (NO2), *sulfur dioxide* (SO2), *ozone* (O3), and *particulate matter* (PM10). These pollutant concentrations are used to calculate the air quality index (AQI). AQI is a commonly used indicator defined by the United States Environmental Protection Agency (EPA) for public use of the air quality conditions. In order to calculate AQI for a particular location, an indicator value I is calculated for each of the observed pollutant concentrations (CO, NO_2_, SO_2_, O_3_, and PM_10_) using Eq. (1) (Mintz 2012).

1 $$ I=\frac{I_{\mathrm{high}}-{I}_{\mathrm{low}}}{C_{\mathrm{high}}-{C}_{\mathrm{low}}}\left(C-{C}_{\mathrm{low}}\right)+{I}_{\mathrm{low}} $$

Where *I* is the air quality index, *C* is the pollutant concentration, *C*_low_ is the concentration breakpoint which is less than or equal to *C* and *C*_high_ is the concentration breakpoint that is greater than or equal to *C. I*_low_ and *I*_High_ are the index breakpoints corresponding to *C*_low_ and *C*_high_, respectively. *I*_low_, *I*_high_, *C*_low_, and *C*_high_are extracted from the EPA’s table of breakpoints (Mintz 2012). After calculating all indicators for each location, the maximum indicator value is considered as the AQI in a given time. According to EPA’s table, AQI is then classified in seven categories.

Standard AQI defined by EPA is used by the Air Quality Control Company of Tehran. Therefore, the air quality monitoring stations measure the pollutant concentrations which are used for AQI calculation. In this study, AQI and its corresponding classes are used as the target information of interest.

**Fig. 2:**
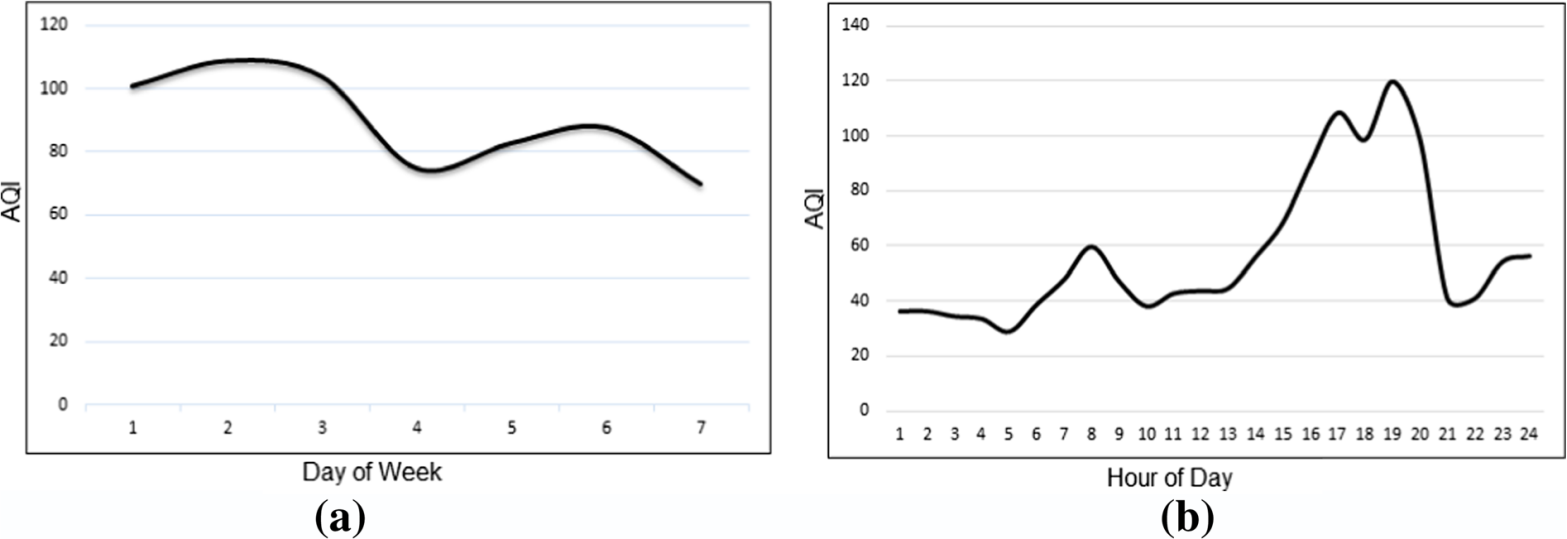
Trend of mean AQI of 6 months period during the week days (**a**) and day hours (**b**) in Tehran

In addition to data of pollution, *days of week* and *hours of day* are considered as the two other effective parameters. Consideration of the mean values of AQI for all the pollutants of 6 months’ time period shows interesting trends during the week days and day hours (Fig. 2a, b**.** Low traffics on weekends lead to lower AQI, and pollution levels increase in the first days of the week. As can be seen, air pollution in Tehran reaches its peaks between 6 to 10 A.M and 15 to 21 P.M.

### Traffic and the terrain data

Three spatial parameters including the traffic, elevation, and surface curvature are considered to monitor the spatial distribution of air pollution. These are briefly described as follows.

#### Traffic

**Fig. 3:**
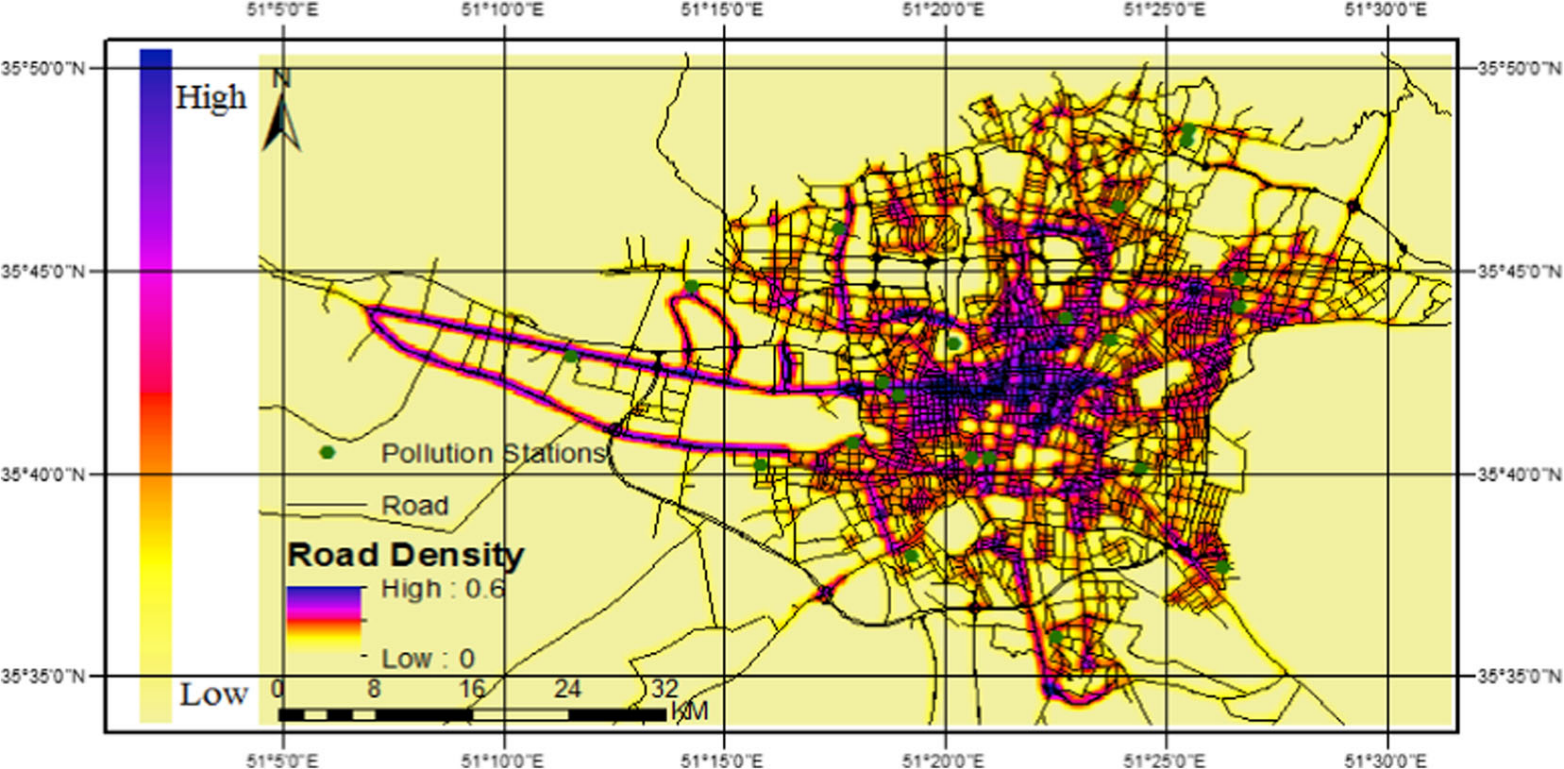
Road density map resulting from application of kernel density estimation with a bandwidth of 300 m in Tehran

Traffic is a major source of air pollution in urban areas (Halek et al. 2004). There is a significant relationship between the traffic-related pollutants and distance to the roads (Barzyk et al. 2009). Therefore, because of the lack of reliable spatial information about the traffic, in this study, air pollution caused by traffic is assumed to be a function of distance from roads. Using the kernel density estimation approach, a raster indicating the density of surrounding roads is created. By considering the maximum distance of 300–500 m (Barzyk et al. 2009) for impacts of roads on air pollution and geographical and wind direction conditions of Tehran, distance of 300 m is selected as the maximum effective bandwidth. Also, roads are weighted by their width and type. Although the non-directional distance function shows deficiencies such as not considering the directionality and complexities of the interactions between the space, wind, and pollution, but it has been preferred because of its simplicity and ease of use. Figure 3 demonstrates the resulting raster output from application of the kernel density approach. Dark areas highlight areas with higher densities of road segments.

#### Elevation

With an average altitude of 1190 m above the sea level and 700 m altitude difference between the lowest and highest locations, Tehran shows considerable elevation variations. Elevation continually increases from south to north. Due to the significant differences in elevation among the various districts, weather conditions are quite different in the northern highlands and the flat southern and central areas. Therefore, topographic conditions show considerable influences on the air pollution patterns (Zheng et al. 2013). Also, Tehran contains seven hills with elevations higher than those of the surrounding areas. Empirical observations show that air quality over the hilly regions is better than those of the neighboring areas. In order to address the effects of elevation on each point, this study utilizes the local mean height along with the point height. The local mean height of each point is calculated within a circular area with an arbitrarily defined radius of 2500 m. Then, point height and its difference with the local mean height of each point are used as the input parameters for pollution prediction.

#### Curvature

Terrain attributes can have important influences on the levels of air contamination in urban areas. Polluted air can be trapped in concave areas, and contamination can be wiped off by the wind in convex areas. Thus, convexity and concavity characteristics of surfaces are employed as the important parameters in this study. Convexity of a landscape is calculated using Eq. (2) (Jenness 2010).

2 $$ \mathrm{General}\ \mathrm{curvature}=-2\left(r+t\right) $$

Where *r* and *t* are the second derivatives of elevation in *x* and *y* directiosns, respectively. In order to calculate curvature, a 3-cell by 3-cell moving window is used, and the curvature is calculated from nine raster cells in the window. Convex and concave surfaces respectively have positive and negative values, and general curvature value near zero indicates a flat area.

### Meteorological data

Five weather stations in Tehran have been established to measure, record, and report various meteorological parameters. National Meteorological Organization is responsible for meteorological data. Wind direction and speed, cloudiness, temperature, pressure and relative humidity collected by these stations are the most important meteorological parameters affecting the urban air quality (Kurt et al. 2008). Thus, these parameters are also fed to the developed air pollution prediction system.

### Development of the dynamic air pollution forecasting system

**Fig. 4:**
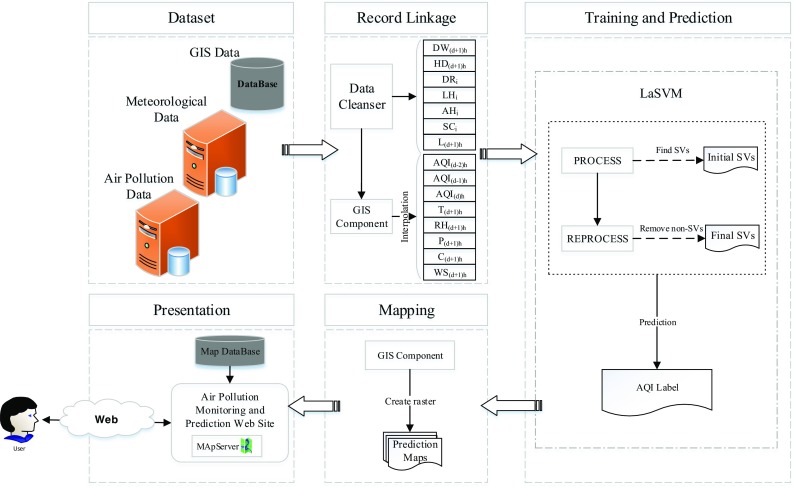
Outline of the developed air pollution forecasting system

An overview of the proposed online system for air pollution prediction is illustrated in Fig. 4. Data of pollutant concentration, weather conditions, and spatial parameters are dynamically collected from different sources and preprocessed as discussed in “Parameters, dataset, and data preparation” sub-section. In order to use the collected data for training, data cleanser component converts data into a specific structure to be fed to the LaSVM module. The LaSVM module utilizes this data structure to train a prediction model. Using the prediction model, LaSVM module predicts the AQI label of the air pollution monitoring stations for the next 24 h. The predicted AQI is then fed to GIS component to create prediction maps using an interpolation method. The output prediction maps are stored in the map database. When a new request for air quality prediction at a specific time and location is sent by users, the related maps are retrieved from the database and presented to the user via an air quality monitoring and prediction website. The process is repeated as a new set of data receives from the monitoring stations.

In order to dynamically train the online system and predict the AQI label, the proposed system utilizes an online algorithm based on the SVM. SVM is a binary classifier derived based on the statistical theory (Vapnik 1998) for classification and regression analysis. In a linear condition, SVM constructs optimal hyperplanes to separate the members of two classes while maximizing the distance between the closest samples of classes (Vapnik 1998). However, in most real-world problems, datasets may not be exactly modeled into the linearly separable partitions. To handle the non-linear cases, kernel functions are used to map the input data to a higher-dimensional space (Haifeng et al. 2009). The mapped data in the new space would be linearly separable (Yu and Kim 2012).

Given a training set of data, {*x*_*i*_, *y*_*i*_}, *i* = 1, …, *l*, *y*_*i*_ ∈ {−1, +1}, *x*_*i*_ ∈ *R*^*d*^(the space of *d* dimension), where *x* denotes the input data, called vectors, and *y* is the corresponding labels, suppose there exists separating hyperplanes which separate the samples with positive labels from those of the negative labels. The closest data points to the hyperplanes are defined as the support vectors and distance between the closest positive and negative samples is known as the margin.

By defining *b* as the bias, in the case of nonlinear condition, the optimization problem can be formulated as the Eqs. (3) and (4) (Burges 1998)^1^:

3 $$ f(x)=\mathit{\operatorname{sign}}\left\{{\sum}_{i=1}^l{\alpha}_i{y}_ik\left({x}_i,{x}_j\right)+b\right\} $$

4 $$ \mathrm{Subject}\ \mathrm{to}\ \mathrm{the}\ \mathrm{constraints}:{\sum}_{i-1}^l{\alpha}_1{y}_1=0\kern0.5em and\kern0.5em \le {\alpha}_1\le C\kern0.5em for\; all\;i $$

Where *K*(*x*_*i*_*x*_*j*_) is the kernel function and *α*_*i*_denotes the Lagrange’s multiplier. The coefficient *α*_*i*_ can be obtained by solving Eq. (5). The closest data points to the hyperplanes with non-zero coefficients *α*_*i*_≠ determine support vectors. The other samples *α*_*i*_ = 0 are far from the hyperplanes and have no impact on the construction of the hyperplanes.

5 $$ \operatorname{Maximize}\;{\sum}_{i=1}^i{\alpha}_i-\frac{1}{2}{\sum}_{i=1}^{;}{\sum}_{j=1}^l{\alpha}_i{\alpha}_j{y}_i{y}_jK\left({x}_i{x}_j\right) $$

*C*, the regularization constant, controls the trade-offs between decreasing the errors and maximizing the margin (Yeganeh et al. 2012).

In typical practice, SVM classifier requires to receive all training data beforehand. This means that this classifier is trained once using the whole training data. Because of this characteristic, the typical SVM algorithm is not a reasonable solution to address problems such as the online and continuous air pollution prediction, where training data are sequentially provided. Because, whenever new samples are provided, the algorithm must be re-trained using all the available data (including old and new coming data) (W. Wang et al. 2008). To address this deficiency, an online SVM algorithm named LaSVM which is the outputs of the recent efforts for applying online algorithm rationally to the typical SVM method, has been used in this study (Rüping 2001; Syed et al. 1999; Wang et al. 2007). LaSVM is an online kernel-based classifier which has been developed by Bordes (Bordes et al. 2005). LASVM, on the contrary to SVM, works in an online setting, where the algorithm dynamically modifies its hyperplanes as new training samples become available. It continuously receives new training samples, finds out the correct label using the trained model at that point of time, and updates its hyperplanes, if necessary, based on the new inserted samples. This characteristic of LaSVM makes it suitable for dealing with big and streaming data.

In order to train the online system, collected data of 5 years from 2008 to 2013 is continually fed to the LaSVM. By using the huge amount of data, LaSVM would be able to model the complex behavior of air pollution distribution in the study area. As shown in training section of Fig. 4, LaSVM uses two steps called the PROCESS and REPROCESS to handle the streaming data. Assume that in (*i*-1)^th^ step, the online algorithm has found a set of support vectors using the current samples and the margin conditions are satisfied. When a new point *x*_*i*_ is added, the PROCESS phase investigates if *x*_*i*_ can be considered as a support vector. If *x*_*i*_is defined as a new support vector, coefficients (α) of other points are updated. Updating may change the coefficient of some of the support vectors to zero. The REPROCESS phase, which starts after the PROCESS phase, finds those support vectors which their coefficients were changed to zero. These samples which do not have impact on the training anymore, are no longer considered as the support vectors. After these steps, the hyperplanes are recalculated by considering the new support vectors. Thus, the algorithm is continually adjusted as new training samples become available (Bordes et al. 2005). Next training step is completed using only the support vectors extracted from the last steps and the newly inserted samples. From now on, the newly collected samples from the monitoring stations are dynamically fed to LaSVM and the algorithm is updated hourly as new sets of data are received. In fact, LaSVM behaves as a function *f*(*x*) which describes relationships between the effective input parameters and the predicted AQI class. In this study, the function is defined by Eq. (6) in which *d* and *h* indices denote the day and hour, respectively. Output of this function is the air quality label for a given location in a given time.

6 $$ {L}_{d+1}^h=f\left({AQI}_d^h,{AQI}_{d-1}^h,{AQI}_{d-2}^h,{T}_{d+1}^h,{RH}_{d+1}^h,{P}_{d+1}^h,{CC}_{d+1}^h,{WS}_{d+1}^h,{DW}_{d+1}^h,{HD}_{d+1}^h,{DR}_i^h,{LH}_i^h,{PH}_i^h,{SC}_i^h\right) $$

Where *d* + 1 is the time of prediction and the first three items respectively are the AQI values of the processing day and the last 2 days. Predicted values of temperature (T), relative humidity (RH), pressure (P), cloud cover (CC), and wind speed (WS) corresponding to the day and hour of prediction construct the next array items. Day of week (DW) and hour of day (HD) are also entered to the sample array. The next four items are distance from major roads (DR), local mean height (LH), point height (PH), and surface curvature (SC) as described in previous sections. Corresponding to each sample, there is a label (L) which introduces the class of the AQI at prediction time for the point of interest.

Once input parameters of each point are known, its AQI label can be predicted by the updated LaSVM using the function *f(x).* These points may be either the monitoring stations or any arbitrarily selected location within the city boundary. If the interested point is a monitoring station, all parameters are available in the databases; otherwise, the required parameters should be calculated. For this purpose, the study area is covered by a grid with cell size of 100 by 100 m. Using the GIS component, AQI of the last 3 days and meteorological data are interpolated for each cell using the Inverse Distance Weighting (IDW) method. In order to create pollution maps, the interpolated values are used for prediction of the AQI class for each cell, and the predicted values are used for creating pollution maps. The prediction maps are created hourly as LaSVM is updated by receiving new pollution and meteorological data from monitoring stations. The output maps are stored in a map database. By receiving a request for prediction from the users (prediction section in Fig. 4), location and time (day and hour) of the point or points of interest are sent to the system to be used for retrieving related pollution maps. In order to visualize the output maps, Open Source MapServer along with OpenLayers client library are implemented in this study.

Selection of the algorithm parameter C and the type of kernel function and the corresponding parameters is a vital step in applying SVM for real-world problems. In fact, the classification accuracy depends on the proper selection of these parameters (Burges 1998). The most common kernel functions for consideration are the Radial Basis (Gaussian), polynomial, and linear functions (Hsu et al. 2003; García Nieto et al. 2013; Juhos et al. 2008; Haifeng et al. 2009). In order to determine the best kernel function, performance of the linear, RBF (Gaussian), and polynomial (Degrees 2 and 3) functions were compared. The results, showed that RBF is the most efficient kernel function for this task (Table 1).

**Table 1:** Comparison of the performance of LaSVM, using different kernel functions

Kernel function	Accuracy	RMSE	*R*-squared
RBF	0.71	0.54	0.81
Polynomial (degree 2)	0.61	0.635	0.696
Polynomial (degree 3)	0.33	0.81	0.25
Linear	0.56	0.642	0.672

The RBF kernel on two samples ***x*** and ***x*’**, represented as the feature vectors in some input space, is defined as Eq. (7):

7 $$ {K}_{RBF}\left(x,{x}^{\prime}\right)=\exp \left[-\gamma {\left\Vert x-{x}^{\prime}\right\Vert}^2\right] $$

Where *γ* is the RBF kernel parameter.

Grid search is applied to select the best parameters for the algorithm, *C* and *γ*, using the leave-one-out cross-validation approach (Cawley and Talbot 2004) on the training set. In this study, values of 2 and 0.0019 were obtained for *C* and *γ*, respectively.

Because of acting as a binary classifier, LaSVM may not be directly used for a multiclass problem. In order to perform a multiclass classification using a binary classifier, multiple binary classifiers are composed to simulate a multiclass classifier (Hastie and Tibshirani 1998). One-against-one and one-against-all strategies can be used to split each multiclass classification into a series of binary classifications. In this study, the one-against-all strategy has been chosen to generate M-Class classifiers (M indicates the number of classes). Each binary classifier separates one class from the rest of the classes. For 7-AQI classes of standard EPA’s definition, 7-binary LaSVMs are constructed in this study. Each LaSVM classifier is trained to separate a given class from the other classes. In order to classify a new data point, the corresponding class label of the LaSVM classifier which generates the largest value is selected. (Vapnik 1998) provide more information about the multiclass classification.

## Results and discussion

To develop an online system to dynamically predict the air quality, the proposed algorithm should use the least possible processing time while preserving the accuracy. To this purpose, prediction results of the LaSVM are compared to those of the typical SVM algorithm. The main reason for selection of the SVM for comparison is its similarity to the LaSVM and the reliability of its results as compared to the conventional statistical methods (Lu and Wang 2005; Luna et al. 2014; Yeganeh et al. 2012). In this respect, collected data of 5 years from 2008 to 2013 are used to train both algorithms. Comparison between the SVM and LaSVM is limited to using the training data from 2008 up to when due to the high volume of the input data the SVM crashes. *Accuracy* (Hsu et al. 2003), *root mean squared errors* (RMSE), and *regression coefficient* (R^2^) (Yeganeh et al. 2012) as respectively defined in Eqs. (8), (9), and (10) are used for evaluation and comparison of the results.

8 $$ \mathrm{Accuracy}=\mathrm{number}\ \mathrm{of}\ \mathrm{values}\ \mathrm{which}\ \mathrm{are}\ \mathrm{correctly}\ \mathrm{predicted}/\mathrm{total}\ \mathrm{number}\ \mathrm{of}\ \mathrm{test}\ \mathrm{data} $$

9 $$ \mathrm{RMSE}=\sqrt{\frac{1}{n}{\sum}_{i=1}^n{\left|{Y}_i-{Y}_i^{\ast}\right|}^2\kern0.5em } $$

10 $$ {R}^2=1-{\sum}_{i=1}^n{\left({Y}_i-{Y}_i^{\ast}\right)}^2/{\sum}_{i=1}^n{\left({Y}_i-\overline{Y}\right)}^{2\kern3em } $$

Where in Eqs. (9) and (10),${Y}_i^{\ast }$ and *Y*_*i*_ are respectively the predicted and observed values. $\overline{Y}$ denotes the mean of observed data in Eq. (10). In addition to the above mentioned criteria, processing time requirements are also considered as one of the evaluation parameters. Since air quality is dynamically predicted, therefore, reduction of the processing time for online applications is an important parameter for dealing with the big streaming data. Finally, performance of the LaSVM has been evaluated using the independent test data and the resulting output prediction map is presented as a demonstrative example.

### Processing time

**Fig. 5:**
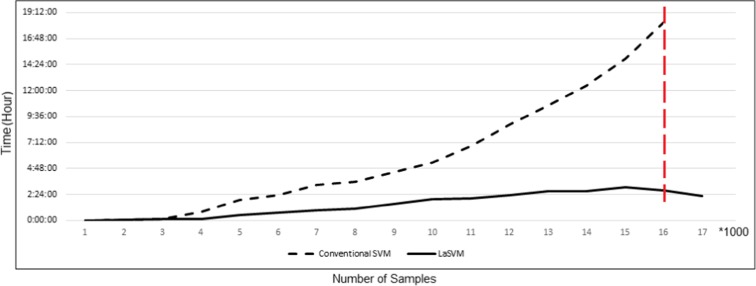
Comparison of the required training times (vertical axes) for the LaSVM and SVM algorithms as a function of sample size (horizontal axis in units of 1000)

The required processing times as a function of training sample size for SVM and LaSVM algorithms are illustrated in Fig. 5. Although training times are nearly similar for both algorithms at the beginning of the training, increase of the sample size leads to exponential growth of the processing time for SVM. With increased number of samples to thousands, addition of even one new sample leads to retraining times of hours for the SVM. Particularly in this study, when the number of training data reaches 16,000, retraining of SVM requires more than 16 h. The exponential growth of the processing time of SVM is due to its use of the all available data for re-training after adding new samples. So, it is obvious that the typical SVM is not capable of being used for online prediction of urban air pollution. Lower increase in the processing time of LaSVM is due to its smarter working principles for selecting smaller numbers of samples including only the previously extracted support vectors and the new sample data.

**Fig. 6:**
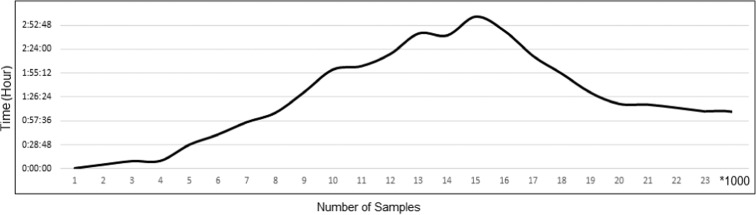
Required training times of the LaSVM algorithm (vertical axes) as a function of wider ranges of sample sizes (horizontal axis in units of 1000)

**Fig. 7:**
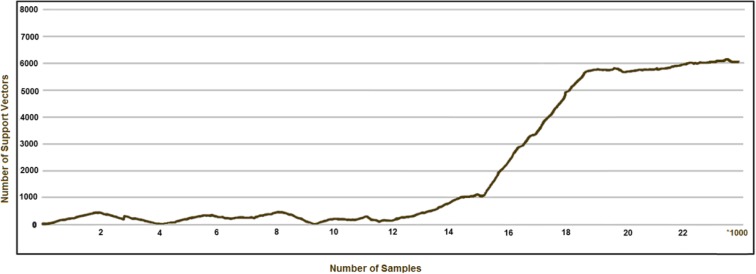
Number of support vectors used by the LaSVM algorithm during the training phase with different sample sizes (in units of 1000)

The trend of time consumption in LaSVM training with respect to larger numbers of training samples is indicated in Fig. 6. In spite of the increasing trends of training time at the smaller numbers of samples, the processing time of LaSVM algorithm starts to significantly decrease after adding about 15,000 samples. The decrease of the processing time coincided with the increase in the number of support vectors (Fig. 6**)**. In fact, reduction of the processing time happens when an adequate number of support vectors is found by the LaSVM, and appropriate separating hyperplanes are constructed. This situation leads the number of support vectors to remain almost constant. Stability of the number of support vectors leads to relative stabilization of the processing time. As shown in Fig. 6, the processing time starts to stabilize after the sample size of 19,000. Number of support vectors used in each step of the training is shown in Fig. 7. The relative stability of the number of support vectors as shown in Fig. 7 may be attributed to equality of the added and removed support vectors, during the PROCESS and REPROCESS phases and representativeness and validity of the constructed hyperplanes which are not violated by inserting the new training samples. However, the stability of the number of support vectors leads to stability and consistency of the online algorithm.

### Accuracy and precision

**Fig. 8:**
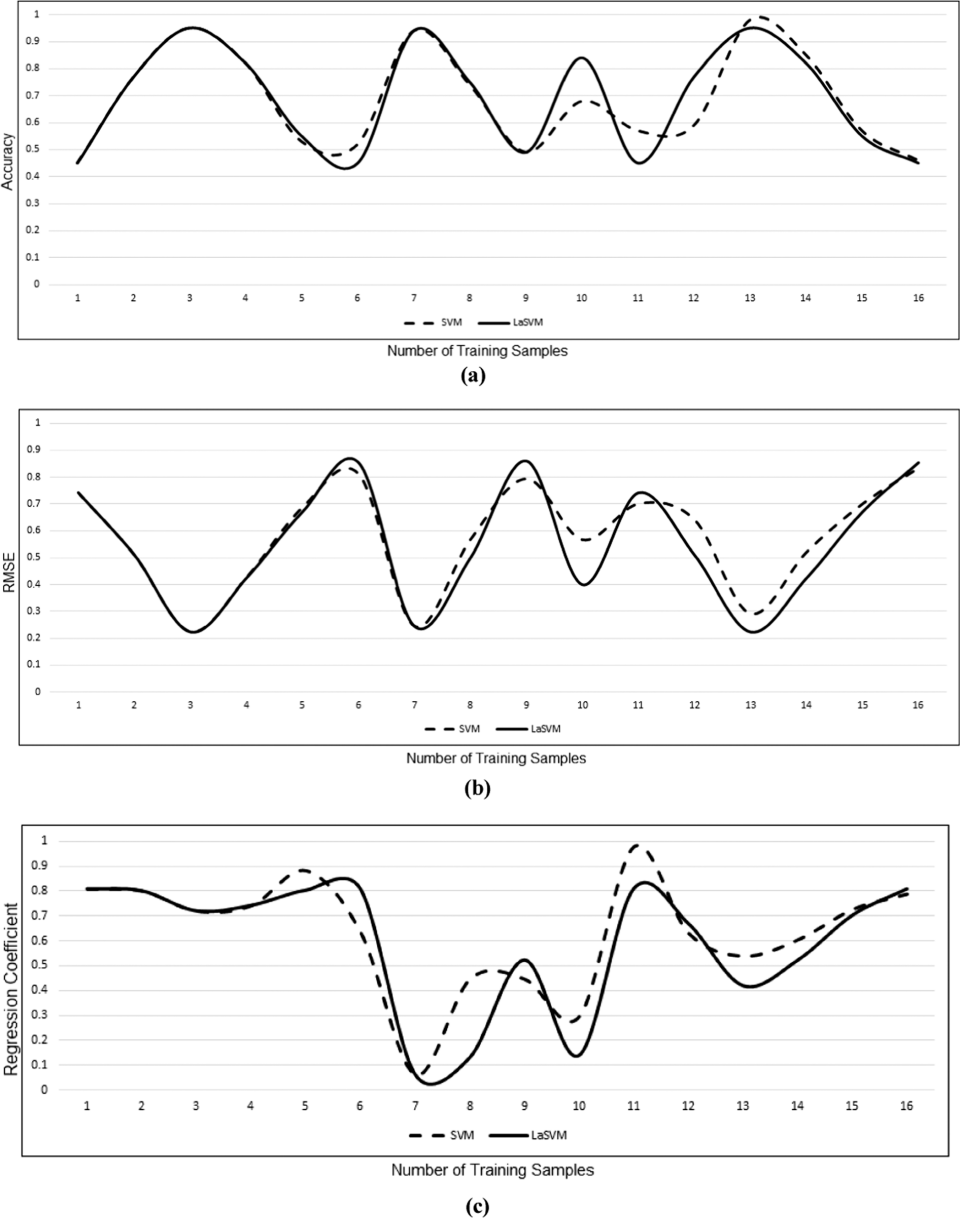
Comparison of the accuracy (**a**), RMSE (**b**), and regression coefficients (**c**) of SVM and LaSVM as a function of training sample size (in units of 1000)

**Fig. 9:**
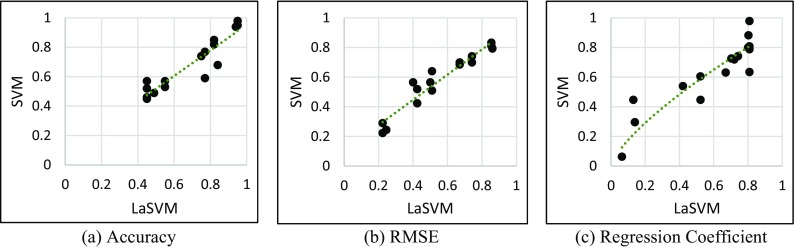
Scatter plots of the relations between the accuracy (**a**), RMSE (**b**), and regression coefficients (**c**) of the SVM and LaSVM

Three indicators of output performance including the accuracy, RMSE and regression coefficients for the SVM and LaSVM are almost the same at early stages of the training (Fig. 8a). However, removal of the non-support vectors from LaSVM algorithm leads to a slight difference between the accuracies of the LaSVM and SVM. Despite these trivial differences, after finding an adequate number of support vectors and definition of the representative hyperplanes, the accuracy of LaSVM is almost similar to the accuracy of the SVM. The same behavior can be seen in RMSE and regression coefficient diagrams (Fig. 8b, c). Average difference of accuracy, RMSE and regression coefficients of LaSVM and SVM for varying training sample sizes ranging from 1000 up to 16,000 are 0.041, 0.046, and 0.17 respectively. The scatter plots displaying correlations between the accuracy, RMSE and regression coefficients of the SVM and LaSVM are illustrated in Fig. 9. The presented scatter plots show that there is a high correlation between the result of SVM and LaSVM, which prove that the proposed system can achieve the SVM accuracy. Numerical results of the correlation coefficients of RMSE, accuracy, and regression coefficients between the SVM and LaSVM are also presented in Table 2.

**Table 2:** Correlation coefficients (*R*^2^) of the accuracy, RMSE, and regression coefficients of SVM and LaSVM

Accuracy	RMSE	Regression coefficient
0.86	0.92	0.7

Regarding the fact that comparison between the SVM and LaSVM is based on the limited number of training samples, up to when the SVM stops working due to the high processing time. Results of independent train and test of the LaSVM using the 5 year training data (2008–2013) and data of 2014 as the test are presented in Table 3 Average accuracy is calculated using Eq.11 as follows.

11 $$ AverageAaccuracy= sum\  of\ the\ accuracy\ column/ number\ of\ classes $$

**Table 3:** Overall and average accuracy, RMSE, and regression coefficients of the LaSVM algorithm for online processing of test data of the year 2014

Overall accuracy	Average accuracy	RMSE	*R*-squared
0.71	0.52	0.54	0.81

**Fig. 10:**
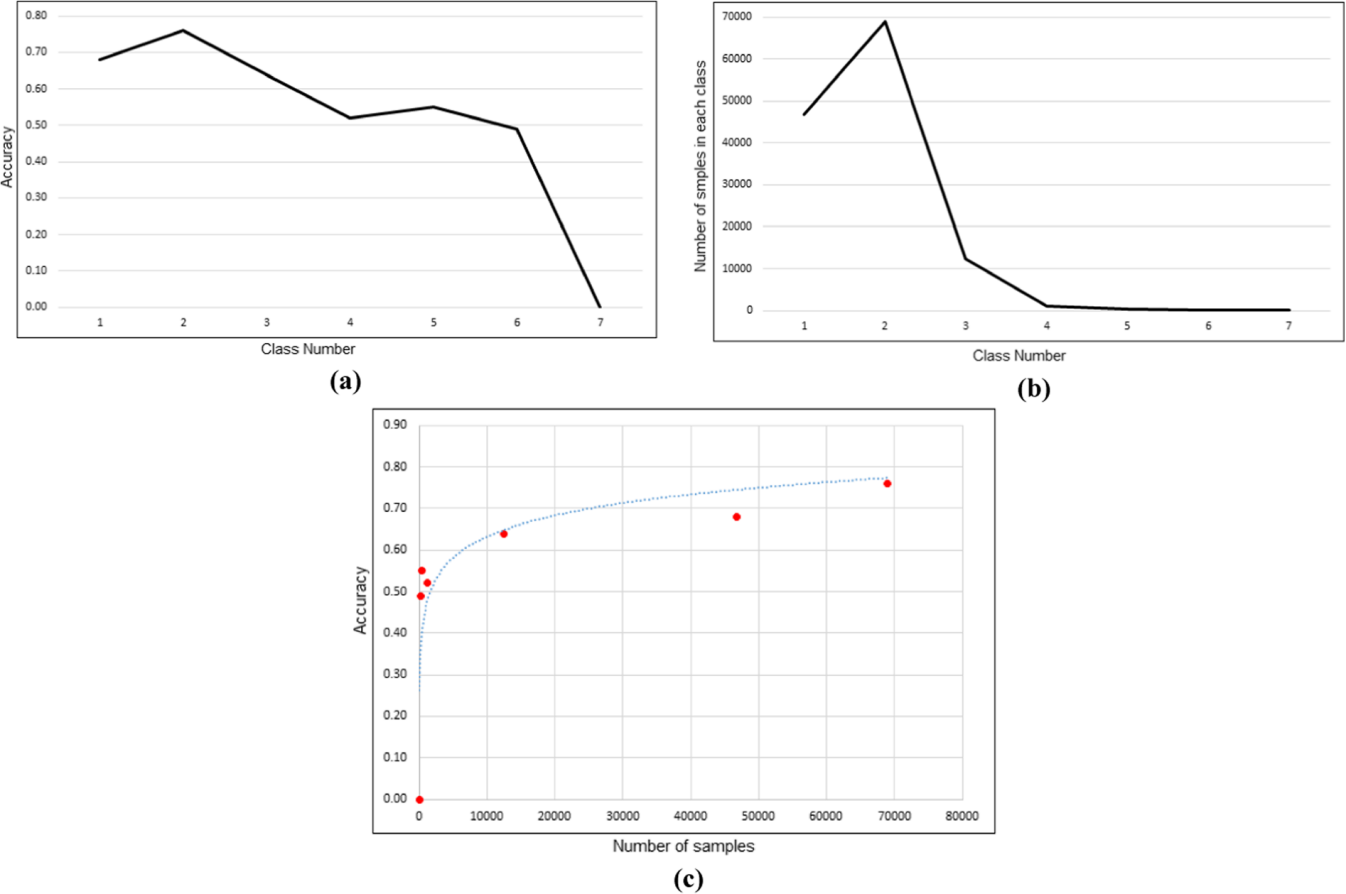
Performance indicators of the LaSVM including the accuracy (**a**), number of samples **(b**), and relationships between the accuracy and number of samples (**c**) for test data of year 2014

**Fig. 11:**
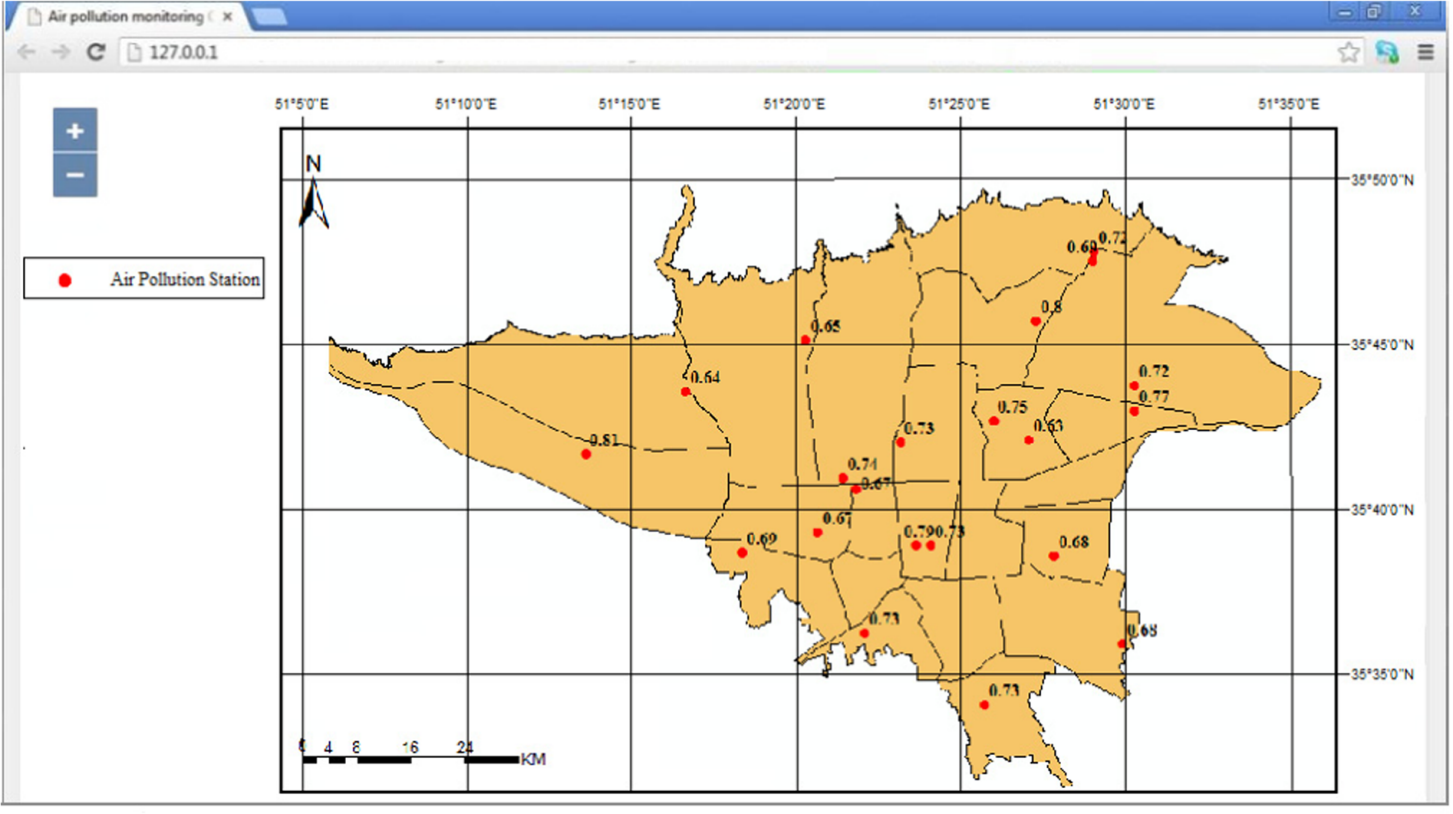
The accuracy of AQI prediction for 21 pollution monitoring stations for data of the year 2014

Table 4 highlights the confusion matrix, accuracy, and precision of the LaSVM for prediction of seven AQI classes for test data of the year 2014. Accuracy and precision columns show the accuracy and precision of each class. Accuracy and number of samples of each class and the relationship between the accuracy and number of samples are presented in Fig. 10. As shown in Fig. 10**c**, accuracy shows a nonlinear relationship with the number of samples. It seems that small sample sizes up to 1000 results in an unacceptable accuracy. The proposed algorithm provides more reliable results for classes with sufficient numbers of samples, whereas the accuracy is lower for classes (5 and 6) with smaller numbers of samples and class 7 cannot be predicted by the algorithm due to scarce number of samples in this class. The accuracy of the predicted AQI for air pollution stations using the 1-year test data is presented in Fig. 11. It should be mentioned that some of the observed errors may be attributable to relying on the non-directional and simple distance-based kernel function for modeling the spatial dependencies between the pollution rates and road densities. Effective consideration of the wind speed, frequency, and direction and their integration with distance functions is expected to increase the performance of predictions.

**Table 4:** Confusion matrix for prediction of seven AQI classes by the LaSVM for data of the year 2014

Predicted observed	1	2	3	4	5	6	7	Sum	Accuracy
1	31,906	13,844	867	49	80	70	0	46,816	0.68
2	14,913	52,357	1574	22	77	9	0	68,952	0.76
3	1178	3254	7938	9	21	10	0	12,410	0.64
4	147	184	217	625	10	2	0	1185	0.52
5	10	121	44	7	219	0	0	401	0.55
6	9	23	27	0	30	85	0	174	0.49
7	11	43	8	0	0	0	0	62	0
Sum	48,174	69,826	10,675	712	437	176	0	130,000	
Precision	0.66	0.75	0.74	0.88	0.5	0.48	0

### Prediction for a specific time

**Fig. 12:**
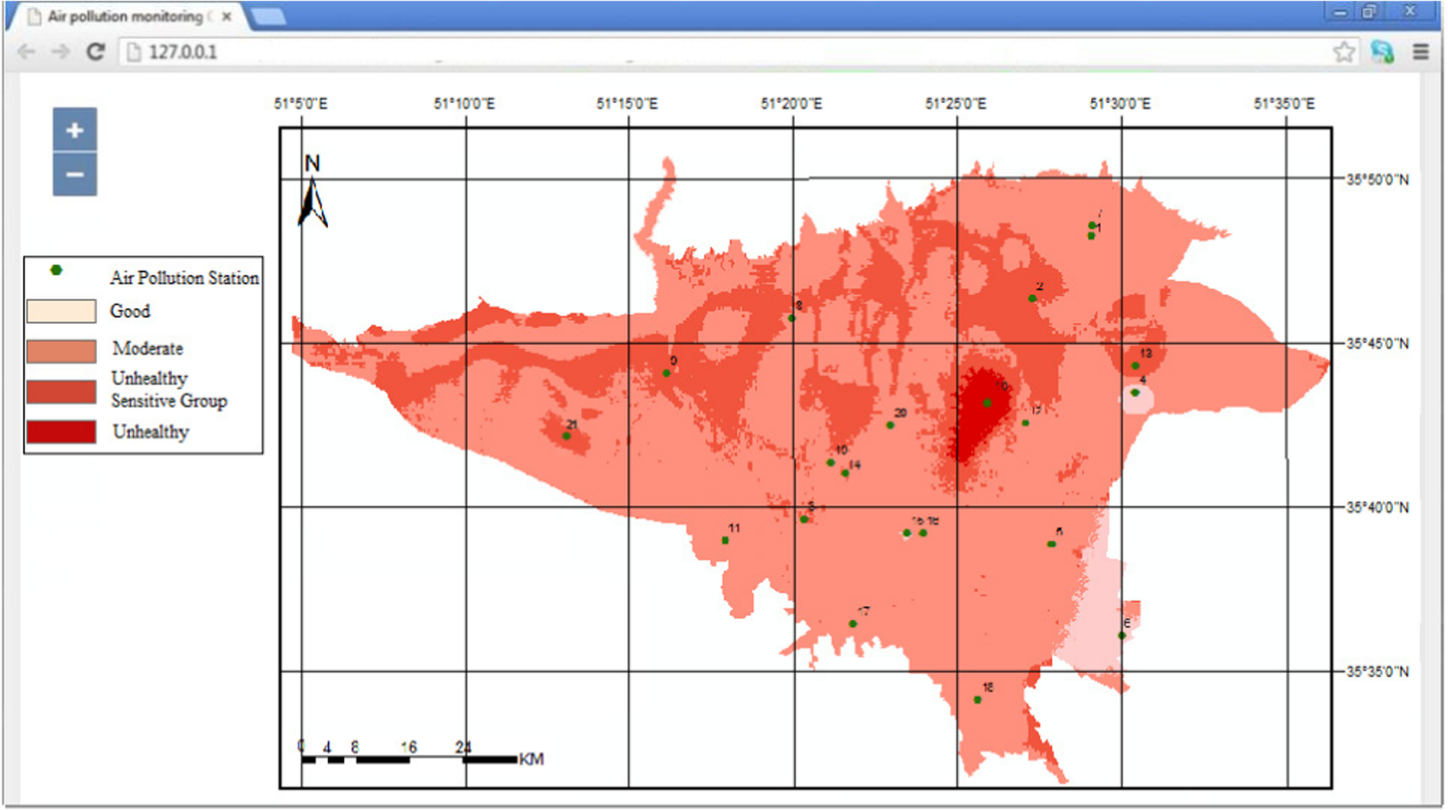
Predicted map of the air pollution by LaSVM for 9 A.M of day 8/11/2012

**Fig. 13:**
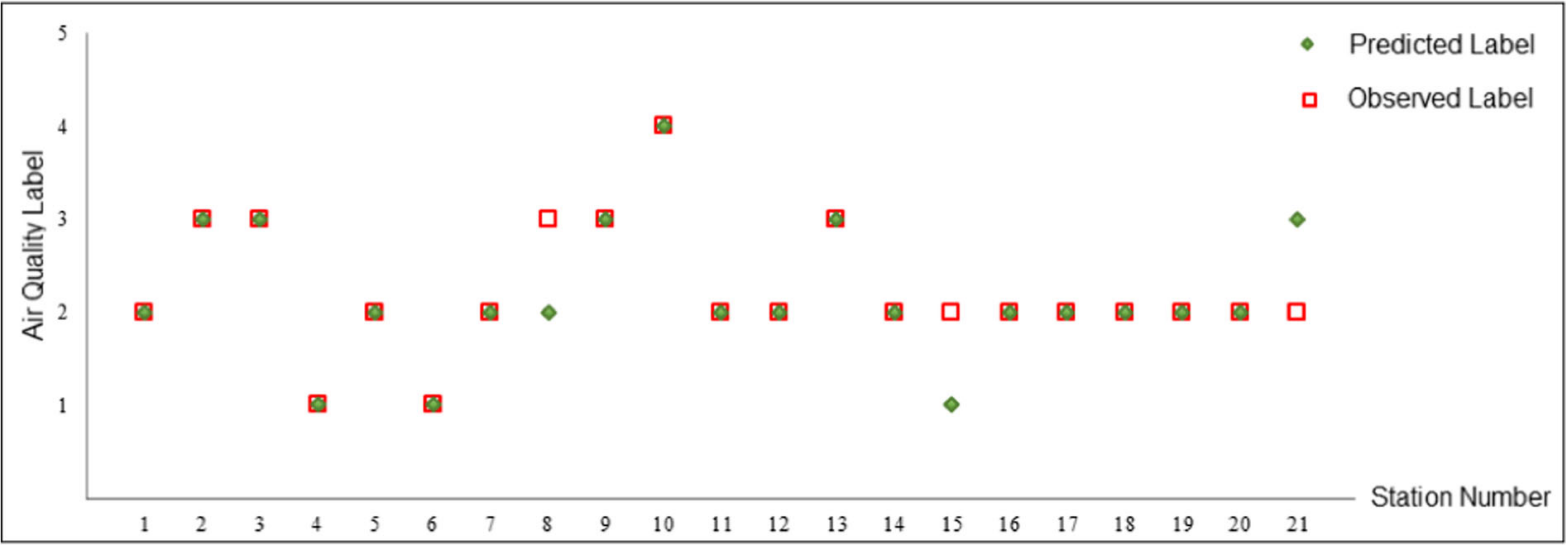
Comparison of the observed and predicted air quality labels by LaSVM, for 21 air pollution stations at 9 A.M of day 8/11/2012

In order to further demonstrate the usefulness of the system for completion of a daily prediction task, air pollution has been predicted for a particular time and day (8/11/2012 at 9 A.M). This day was chosen due to various reports for having high air pollution and warnings for sensitive groups in media. The predicted pollution map is shown in Fig. 12. Comparison of the predicted and observed air quality labels over the pollution stations for 9 A.M of day 8/11/2012 shows that the air quality class for 18 of the 21 stations are accurately predicted (Fig. 13). Map-based output of the online system can be used to highlight the risky areas and provide preemptive warnings for sensitive groups. Also, the output maps can be used for detailed analysis of the spatial distribution of pollution for understanding and improving the air quality state.

## Conclusion and future work

An online air quality prediction system for Tehran based on the LaSVM algorithm has been developed in this study. Because of the capabilities to solve the problem of dealing with big streaming data collected by the air quality and weather stations, the online learning algorithm is able to continuously predict the air pollution. Along with the historical air quality and weather data, this system also utilizes terrain and traffic-related data to spatiotemporally predict the air pollution concentration. Real-time data provided by the monitoring stations along with the geographical data are continuously fed to the algorithm to predict the AQI labels. The prediction maps are hourly produced and can be accessed via a website. Performance of the system has been compared with those of the conventional SVM. Processing time and statistical error estimators including the accuracy, RMSE, and regression coefficients have been used as the performance indicators*.* The advantage of the developed system is that the processing time significantly decreases by removing the nonsupport vector samples in the training step, and without decreasing the accuracy.

The developed system can serve decision makers and the public by providing sufficient information to perform preemptive arrangements for dealing with severe air pollution conditions. By identifying the risky areas and times that air pollution is harmful, some measures such as setting special warnings for sensitive groups can be used to decrease the daily exposure to pollution and outdoor activities. Such solutions can significantly reduce the respiratory and cardiovascular diseases caused by the air pollution.

The proposed online system was able to continuously work with the streaming data of Tehran on a single machine. However, it is possible to improve the performance of the system by dividing its workload among multiple processing machines, using Apache Hadoop parallel computing framework. Via Hadoop, the input data can be partitioned into different parts; each part will be saved and processed on a processing node where the support vectors will be extracted, and finally, all extracted support vectors can be used for constructing the model. In this regard, extending the proposed solution for working on Hadoop will be conducted in the future. Another important issue about the proposed system is the imbalanced dataset which does not allow the algorithm to be trained properly for classes with a smaller number of samples. Improving the system to be able to deal with the problem of imbalance dataset is considered as another direction in our future research. We will also examine the feasibility of the proposed online algorithm to separately monitor the behavior of each pollutant concentration, particularly CO2 and PM10. Comparing the proposed method with new ANN methods as well as the deep learning can also be considered as a future direction. In this research, a simple distance-based kernel function has been used to model spatial dependencies between the pollution and road densities. By employing sophisticated functions based on exploration of the relationships between the wind, space, and pollution, we expect to considerably enhance the performance and reliability of the results in the future.

## Footnotes

1 Notation in part “Development of the dynamic air pollution forecasting system” is borrowed from: (Burges 1998)
